# Feasibility study of a screening decision tool for allocating adults into food is medicine programs as an effective approach to target clinical and dietary outcomes among rural and urban adults

**DOI:** 10.3389/fpubh.2026.1783076

**Published:** 2026-04-23

**Authors:** Alison Gustafson, Carolyn Lauckner, Joshua L. Bush, Emily Dimond, Carolina Morales Serrano, Christa Mayfield, Elizabeth T. Anderson Steeves

**Affiliations:** 1 Department of Dietetics and Human Nutrition, Martin Gatton College of Agriculture, Food and Environment, University of Kentucky, Lexington, KY, United States; 2 C.S. Mott Department of Public Health, College of Human Medicine, Michigan State University, Flint, MI, United States; 3 Kentucky Injury Prevention and Research Center, College of Public Health, University of Kentucky, Lexington, KY, United States; 4 Center for Nutrition and Health Impact, Omaha, NE, United States

**Keywords:** dietary intake, food insecurity, food is medicine, hypertension, screening tool development

## Abstract

**Objective:**

Evaluate feasibility (primary), clinical, and cost outcomes (secondary) associated with the use of a screening decision tool for allocating rural and urban adults with hypertension into in a food is medicine (FIM) program.

**Design:**

Participants were allocated to receive either medically tailored meals or grocery prescription (Rx), based on a user-centered designed screening decision tool. Medically tailored meals (MTM) provided 5 meals per week for 12 weeks. The grocery Rx program provided $100 each month for 3 months to purchase food consistent with guidelines for hypertension. Feasibility outcomes included engagement, dose, and program acceptability. Semi-structured interviews were completed with a subset of 20 participants to obtain feedback on the program. Baseline and post-intervention clinical measures were obtained from electronic medical records. Cost analyses data were collected for the 3-month program among both types of programs.

**Setting:**

Two large hospital systems (one rural, one urban) in Kentucky.

**Participants:**

Adults aged 18–64 with hypertension who screened positively for food insecurity and wanted assistance.

**Results:**

A total of 159 participants referred were enrolled, and 144 participants completed all measures (complete case rate of 91%). The screening decision tool had a match rate of 94%−97% for grocery Rx and 73%−77% for medically tailored meals. 95% of participants (engagement rate) completed all 12 weeks or 3 months of the FIM intervention. The average net promoter score (i.e., if they would refer the program to a friend) was 9.4 out of 10. There were significant changes in systolic and diastolic blood pressure among rural and urban adults receiving the grocery Rx program. Those receiving the MTM program in urban communities also reported reductions in systolic and diastolic blood pressure. Qualitative findings reported that the FIM programs were associated with high levels of satisfaction, helped ease the burdensome cost of food, and improved various aspects of their health.

**Conclusions:**

A screening decision tool is associated with high patient engagement and retention in a FIM program, while also improving blood pressure, dietary intake, and general health status.

**Clinical trial registration:**

NCT07011251 on ClinicalTrials.gov

## Introduction

Across the United States, food insecurity rates have doubled between 2005 and 2021 (1). In addition, millions more report experiencing nutrition insecurity, defined as lack of consistent affordable access to nutritious foods and beverages that promote health (2). Very low food security is defined as “reports multiple indications of disrupted eating patterns and reduced food intake (3, 52)”. Food and nutrition insecurity are associated with increased risk of cardiovascular disease (4), hypertension, and diabetes (5). Those from lower socio-economic statuses experience higher rates of food insecurity and diet-sensitive chronic disease (6–10).

Currently, several federal food programs have aimed to address food insecurity through providing supplemental funds to help families or individuals receive additional food, such as the Farmers' Market Nutrition Program or Congregate Meals Program. The largest federal safety net program is the Supplemental Nutrition Assistance Program (SNAP), which provides on average $183 per month per qualifying individual for food-related purchases (11). While there is a body of research indicating SNAP has been successful at addressing food insecurity, (12–14) SNAP is not explicitly intended to encourage healthy food options that are tailored for specific diet-sensitive chronic disease. Thus, the Food is Medicine (FIM) movement was created to address the complexity of food and nutrition security among food insecure and lower income households (15), while also aiming to improve clinical outcomes (16)

Many states have launched FIM programs through an 1,115 Medicaid Waiver or through In Lieu of Service protocols (17). These food and nutrition services provide medically tailored meals (MTM), grocery prescriptions (Rx), meal boxes, or produce prescriptions for those enrolled in Medicaid and who meet other eligibility criteria. These programs are currently offered in select states and among the insured. Preliminary studies have found that FIM has reduced food insecurity, improved health outcomes, and dietary quality (18–20). Meanwhile, others have found no significant change in health outcomes (21). In general, results thus far have found a reduction in healthcare costs, fewer hospital admissions, and fewer admissions to skilled nursing (15, 22). A key limitation of some of this work is the lack of tailored programs to meet patient needs and preferences (e.g., cooking ability, access to a full kitchen), which might allow greater engagement and improved outcomes. While randomized controlled trials are necessary to answer key questions on dose, duration, and clinical effectiveness (2) there is an equally important question related to designing these programs based on user needs and preferences. Thus, utilizing a screening decision tool to allocate participants into a FIM that meets their needs may be just as effective at engagement and utilization. (23) In some FIM programs, activation of subsidy cards has been below 50% (24, 25), and in healthy food incentive programs, like the US Department of Agriculture Gus Schumacher Nutrition Incentive Program (GusNIP) produce prescription program, only about 65% of the card balances are used (26). These low engagement rates may indicate that there is no one-size-fits-all approach and that offering only one type of program that is not tailored to individuals' needs will not produce the intended outcomes (27).

Recently, the American Heart Association (AHA) launched the Health Care by Food™ (HCxF) program, which is advocating for a user-centered design approach. Our study utilized this framework to develop a screening decision tool to allocate patients to a FIM program based on their preferences and resource constraints, while also balancing the cost of these programs. The aims of this study are to: 1) Determine feasibility of a screening decision tool to match adults to the appropriate food is medicine program; 2) Explore how a screening decision tool allocation may impact process measures of engagement, retention, and usage; 3) Examine how those allocated to two different FIM programs improve upon clinical outcomes of blood pressure and self-report of dietary intake, financial strain, and general self-reported health; 4) Report on cost analyses of the program among participants; and 4) Report on the user experience among participants through collection of qualitative feedback.

## Methods

The data that support the findings of this study are available from the corresponding author upon reasonable request.

We have described recruitment, enrollment, and eligibility in a previous publication in more detail (EAS, accepted for publication, JHPCU 2025). Briefly described below are our screening, referral, and enrollment steps.

### Eligibility criteria

To be eligible to participate in this pilot study, patients had to be between the ages of 18–64, have a diagnosis of hypertension (ICD-10 code I10), and speak conversational English. Only one patient per household was eligible to participate. Participants were recruited over a 45–day period from two large hospital systems in rural and urban communities in Kentucky.

### Screening

Two screening processes, automated or face-to-face clinician-administered, were used based on the current structure and preferences of the health system partners. The automated procedure consisted of the patient receiving an e-mail with instructions to log into their patient portal before check-in and complete the Hunger Vital Signs screener, (28) with responses recorded in the electronic medical record (EMR). The face-to-face procedure consisted of the nurse or clinic staff asking the patient the Hunger Vital Signs screener in the patient room and documenting their response in the EMR.

### Referral

If the eligible patient expressed interest in participating, the healthcare clinic staff would input their relevant contact and eligibility information into a Research Electronic Data Capture REDCap form (29) for the Food as Health team at the University of Kentucky to contact the patient for further enrollment procedures.

### Enrollment into the HCxF program

Staff from the Food as Health program coordinated the enrollment of patients and subsequent delivery of the food package. Referred patients received a welcome message via text and email with a link to the baseline survey and phone number to enroll. Follow-up messages were sent 2 days later to patients not enrolled, after which staff made three attempts by phone to assist with enrollment. Enrollment consisted of informed consent for the pilot study, use of a screening decision tool to match participants to the appropriate program, and a baseline survey.

### Development of the screening decision tool

The study team, consisting of experts in nutrition and social determinants of health, constructed a screening tool that considered user preferences, such as a desire to shop for food independently or prepare meals, while balancing resource constraints such as having reliable transportation to get to a grocery store. The development of the tool drew upon prior research and utilized previously-validated social determinants of health items. (30, 31) The team pilot tested the screening tool among previous FIM patients in rural and urban areas to understand if the tool was sensitive to preferences and constraints. A total of *N* = 31 pilot tested the tool and provided feedback via cognitive interviews, which resulted in the following revisions: 1) removed stigmatizing language, such as ‘prepackaged meals;' 2) asked if participants are *easily* able get to the grocery store rather than just “able.” As one participant noted, she could “*walk to the store on good days, but not always”*; 3) asked participants if they “preferred delivery more than in-person shopping” versus “preferred choosing food on their own in store more than delivery.” The original phrasing seemed to be biased toward in-person shopping. The study team made final edits and tested the revised version among *N* = 8 additional participants (see Supplemental Materials for the final screening decision tool) to ensure the tool was functioning as intended and that no additional issues were reported.

### Screening tool allocation into FIM programs

The team then utilized the screening decision tool to allocate individuals into a Food is Medicine program consisting of either medically tailored meals or grocery prescription programs (in-store or online). These modes of delivery were selected based on evidence from the literature related to clinical effectiveness (2, 32) and the team statewide development of these programs from the past 2 years (17). The following describes the content of the food is medicine options that were offered to participants for 3-months.

#### Medically tailored meals (MTM)

Mom's Meals was the provider of MTM, which consisted of 5 meals per week for 12 weeks tailored to those with hypertension. The meals were ready to eat and only required re-heating. The meal options were low in sodium, saturated fat, and met guidelines from AHA. Ten meals were delivered to participants' homes every other week (6 deliveries) for those that were allocated to this program. Participants could select meals from a menu of options that met dietary guidelines before each delivery.

#### Grocery Rx (urban areas)

Kroger, with a partnership from Soda Health for the grocery prescription reloadable cards, was the provider for this arm and consisted of $100 per month for 3 months. The food options were low in sodium and saturated fat and emphasized fruits, vegetables, and low-fat dairy. As described in previous publications (17), Soda Health, now Evermore, is a health technology solution that allows restrictions to be implemented on their cards, as a healthy benefits debit card. The team works with Soda Health to create an eligibility list of healthy food items to be purchased on the card and restricts other foods for purchase that do not meet dietary guidelines. These options meet criteria from AHA dietary guidelines and were created by Registered Dietitians from the study team and from Kroger Health. The card was sent to participants' homes at the start of the program, and $100 was reloaded onto their card for the remaining months.

#### Grocery Rx (rural areas)

Food City was the provider for this arm and consisted of $100 per month for 3 months in the form of physical vouchers mailed to participants' homes. The food options were the same as described for the grocery Rx (urban). Four vouchers worth $25 each were mailed to participants each month. Participants were encouraged to use the full amount of funds available from the vouchers when shopping as change could not be given for an unused portion of a single voucher.

#### Grocery Rx (online)

Instacart Fresh Funds program was the provider for this arm and has been described previously (17). This online program offers participants a code to be redeemed online to purchase healthy food items that are delivered to their door. Participants needed to have a bank account and funds available in that account in order to use this program. The decision tool took into account this user constraint when allocating patients to various programs and thus only one participant had the capacity to utilize this program. Due to Instacart not being available in some rural areas, grocery Rx online was only available to urban participants for this study.

### Data collection

Patients completed a baseline and post-intervention survey, which asked questions on all process measures (primary outcomes) and self-report on questions related to dietary intake, financial strain, general health, and other key demographic variables. In accordance with the AHA HCxF program, we utilized the common core and preferred measures (33). We also collected data on employment and engagement for comparison to other studies. Table 1. provides details regarding timing and exact measures used. Everything in Table 1. is a core or preferred measure by AHA.

**Table 1:** Outcomes and process measures, instrument, and timing of data collection.

Clinical and self-report outcome measure	Instrument/data source	Timing
Biometrics (blood pressure, primary outcome)	Patient electronic medical record	Baseline, 3-month
Food insecurity (51)	USDA 6-item food security screener	Baseline, 3-month
Nutrition security	1-item Center for Nutrition and Health Impact	Baseline,3-month
Diet quality	DSQ-10	Baseline,3-month
General health status (33)	Single Item: *Would you say that in general your health is excellent, very good, good, fair, or poor?*	Baseline, 3-month
Mobility, self-care, usual activities, pain, depression (33)		Baseline, 3-month
Financial strain	Single Item: I don't have money to pay my bills	Baseline, 3-month
Feas measures		
Net Promoter Score	Single Item “How likely is it that you would recommend the [intervention] program to a friend?”	Post Intervention
Budget impact	Single Item “How did the program affect your budget?”	Post intervention
Engagement (self-report)	Single Item “If you had to pay for this program, would you choose to participate?	Post Intervention
Communication	E-mail, text, and phone calls	3-months
Redemption Rate Grocery Rx	Amount of grocery Rx funds used/amount of funds issued	3-months
Dose MTM	% of meals delivered	3-months
Engagement rate (Objective)	# of enrolled/# that completed the program	3-months
Complete case	# completed program with all measures collected/#enrolled	3-months

Clinic nurses uploaded protected health information (PHI) into our referral portal using REDCap, which is HIPAA authorized for PHI. PHI was uploaded at baseline and post intervention including systolic and diastolic blood pressure (secondary outcome), height, weight, and hypertension medication use. Patient health data was captured from clinic staff as part of standard of care. Baseline data was defined as the systolic and diastolic captured in the past year. Post intervention data was captured within 45 days of completing the intervention. The study team reminded participants to return to their primary care provider for a follow-up visit to capture PHI at timepoint two.

Semi-structured qualitative interviews were also conducted to obtain post-program participant/user perspectives. An interview guide was developed and reviewed by the study team to obtain information related to participant satisfaction with the HCxF program they were matched to, barriers and facilitators to participating, perceived impacts of the program, and any recommended changes. A purposive sampling strategy for the interviews was used to obtain feedback from the various HCxF prescriptions (MTM, grocery Rx card/urban arm, and grocery Rx vouchers/rural arm) with a target sample size of approximately seven participants per group. The grocery Rx online option was omitted from sample due to the small size (*n* = 1). If selected, the participant was contacted by the research team and invited to participate in the interview. Interviews were conducted by trained research staff over the phone and audio recorded with permission. Recordings were transcribed verbatim and reviewed by interviewers for accuracy. Participants received a $50 incentive for completing an interview. Recruitment and interview data collection occurred until saturation was reached, which was defined as identifying no new information during the interview (34). After saturation was reached, an additional four interviews were conducted to confirm saturation, and then data collection was discontinued.

### Analyses

Descriptive statistics with mean, SE, and percentage are reported for demographic variables. T-test and chi-square tests were used to look at changes pre- and post-intervention. Linear regression adjusted for sex, age, hypertension medication use, household size, household income, and race/ethnicity to examine the effect of participation in the FIM on primary and secondary outcomes. GLM was used to assess mean changes in dietary intake for men and women baseline to post intervention. Dietary intake is analyzed separately for men and women because they have different dietary needs and national guidelines. Chi square analyses were used to measure changes for categories in general health, financial strain, and food security status.

### Cost- data analysis

To assess costs associated with the interventions, we included baseline costs that were incurred regardless of the study arm and those that were specific to each arm. Costs related to the training of clinic staff, screening patients for eligibility, referral and enrollment, as well as development and distribution of patient materials are included in the base costs of the intervention. Participant support and assistance efforts were also recorded as total costs, not associated with a specific arm of the study.

### Semi-structured qualitative interview analysis

Thematic analysis (35) was used to identify themes from semi-structured interviews with participants who had recently completed the program. A codebook was iteratively developed by the coding team (CM, ED, and EAS). The initial codebook was developed by having coders complete line by line coding of two information-rich transcripts to identify axial codes. The axial codes were then discussed by the coders, and codes were defined. The codebook was then uploaded into Dedoose (version 9.2.12, Los Angeles, CA: SocioCultural Research Consultants, LLC) Qualitative Analysis software. Two interrater reliability (IRR) training tests within Dedoose were completed. Tests consistently found a kappa statistic >0.6 indicating substantial agreement between coders. After the IRR tests were run and found to be acceptable, the remaining transcripts were independently coded. Throughout the coding process, the coding team met weekly to discuss coding questions/discrepancies, emergent themes, and memos created during the coding process. Themes were identified from the coded transcripts and summaries with supportive quotes were created for each theme.

## Results

### Study diagram

**Figure 1:**
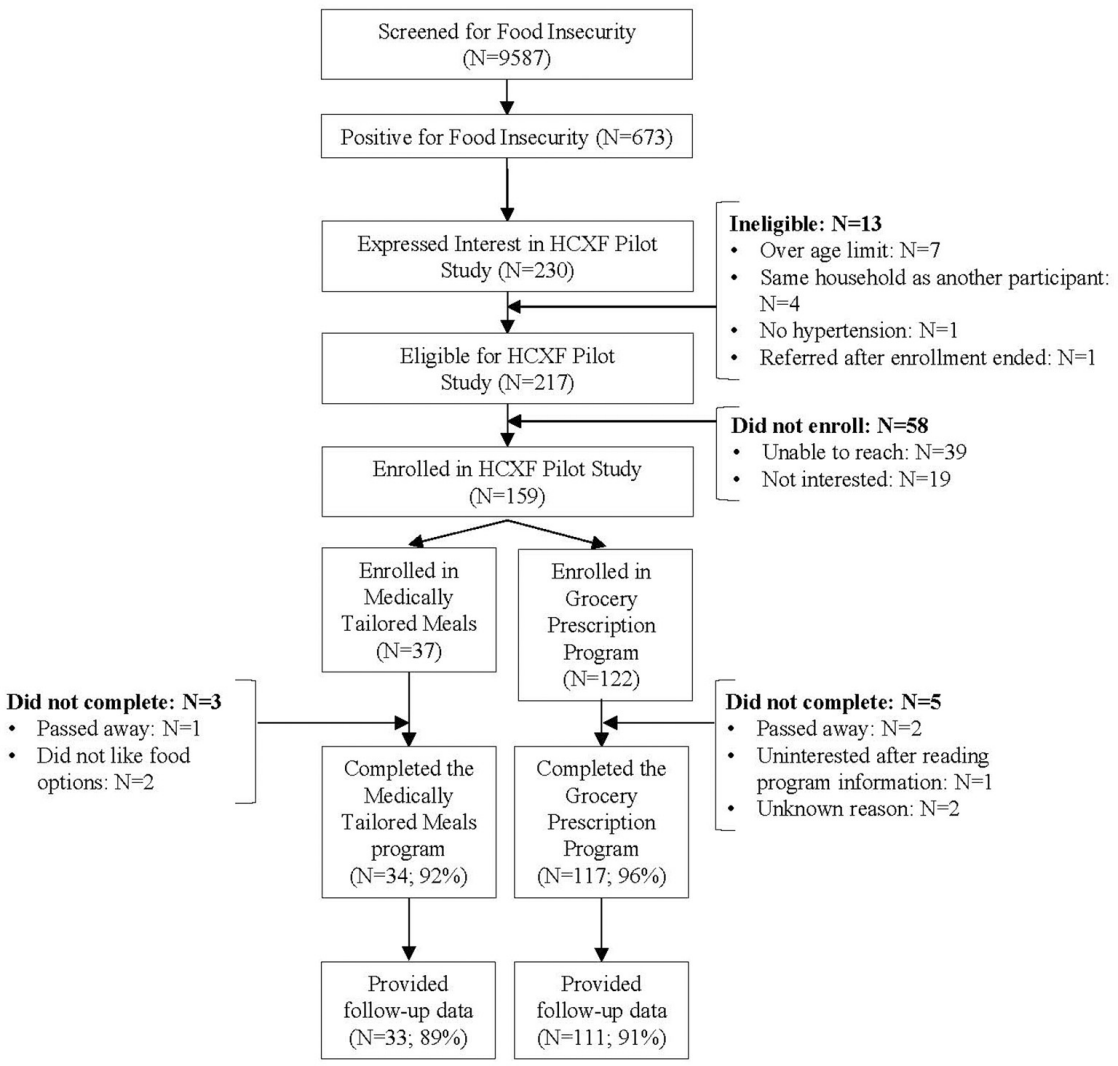
Study flow of the screening, referral, enrollment & engagement process.

Figure 1 provides the study flow from screening, referral, enrollment, and engagement over the entire study period. Of those that were screened for food insecurity (*N* = 9,587) a total of *N* = 673 screened positive (7% 673/9,587). Of the 673 that screened positive *N* = 230 agreed to referral (34% referral rate). Of the 230 a total of *N* = 217 met eligibility criteria. Of the *N* = 217 a total of *N* = 159 completed all enrollment steps (73% enrollment rate 159/217).

Of enrolled participants who completed the program, *N* = 7 did not provide health outcomes at post intervention. Of those, 3 were in the rural grocery Rx arm, 3 in the urban grocery Rx, and 1 in the urban MTM.

### Screening decision tree results

Table 2 provides the key outcomes for how the screening decision tool performed in matching individuals to their need- and preference-based FIM program. The screening tool was effective at matching individuals to the grocery Rx program in-store or online in both rural (94% match rate) and urban settings (97% match rate). However, among those who matched to medically tailored meals, the match rate was lower in both urban (73%) and rural (76%) communities. First, among geographic locations the match rate is the same indicating that the tool was sensitive to geographic location needs. Second, the match rate was lower among MTM with stated reasons of wanting to prepare their own foods, not wanting meals to sit outside, and concerns about allergies. However, the tool was sensitive for matching based on need and preference for the grocery Rx programs. These results suggest further refinement of the tool for screening preferences on cooking, ability/availability to receive and store home food deliveries in a timely manner, and sensitivity to allergies beyond what was asked.

**Table 2:** Screening decision tool outcomes (*N* = 159) and process measure results (*N* = 136 for post survey data collection and *N* = 151 for engagement metrics).

Screening decision tree allocation of participants who enrolled (*n* = 159)		
	% matched to program	% agreed to match
MTM–rural	10.7 (*n* = 17)	76.5 (*n* = 13)
MTM–urban	16.4 (*n* = 26)	73.1 (*n* = 19)
Grocery Rx–rural	44.0 (*n* = 70)	94.3 (*n* = 66)
Grocery Rx–urban	28.3 (*n* = 45)	97.8 (*n* = 44)
Online Grocery Rx–urban	0.6 (*n* = 1)	100 (*n* = 1)
**Reasons for wanting to switch programs after screening tool match include:** Of the 159, 16 patients chose a different program than what they matched to with the screening tool. •11 changed from MTM to grocery Rx with stated reasons including: wanting choice, to shop and prepare their own food; didn't want deliveries to sit outside; and reported allergies •5 changed from grocery Rx to MTM with stated reasons including transportation barriers but the grocery Rx online was not chosen due to participants needing to have an active bank account to utilize the online purchasing functions.	
– Net Promoter Score (*N* = 136, scale 1–10; not at all likely to very likely)	Mean: 9.45 (95% CI 9.21, 9.69); *Indicates high likelihood of telling a friend about the program*
Budget Impact (*N* = 136)	54% were able to buy additional food 54% were able to buy better quality food 49% were able to use money typically spent on food for other needs
Engagement (Self-report) (*N* = 136)	Yes, would pay: 39% No, would not pay: 37%
Communication	On average, participants sent 6 text messages, 1.1 emails, and placed 0.5 phone calls over the study period
Redemption Rate (Grocery Rx) (*N* = 151)	Urban Mean: Month 1: $84; Month 2: $82; Month 3: $76 Median: Month 1: $96; Month 2: $93; Month 3: $95 Rural Mean: Month 1: $83; Month 2: $92; Month 3: $85 Median: Month 1: $98; Month 2: $100; Month 3: $100
Dose MTM (*N* = 151)	99.5% of intended meals were delivered (Of 34 participants enrolled, 33 received all 6 meal deliveries, 1 received 5 deliveries)
Engagement eate	151/159 = 95% completed program
Complete case	144/159 = 91% completed all follow-up measures

### Feasibility measures results

Table 2. provides key results on feasibility outcomes. In general, participants reported a very high net promoter score indicating they were very likely to tell a friend about the program. Close to 40% reported they would pay for this program if it were not free. In addition, our program had a very high redemption rate of close to the full amount of funds being used each month. Almost all those in the MTM completed the program.

### Clinical results

Clinical and self-report measures are shown in Table 3 and Table 4. The average age of participants was 52 years, and a majority were White and had at least a high school degree. There was a high unemployment rate of about 78%, and 50% participation in SNAP. Between baseline and post-intervention across all groups, there was a significant change in SNAP participation, financial strain, and general health status.

**Table 3:** Demographics of study participants and pre/post results across the intervention period.

Demographic Variables	Baseline (*N* = 144) *N (%)*		
Sex			
Female	90 (62.50%)		
Age (years)	52.97	(SD = 9.00)	
Race/ethnicity			
White	119 (82.64%)		
Black or African American	22 (15.28%)		
Hispanic or Latino	2 (1.39%)		
Education level			
Less than HS	42 (29.17%)		
HS Graduate/GED	78 (54.17%)		
College Graduate	24 (16.67%)		
Employment status			
Employed	32 (22.22%)		
Not 15.6-7.5,-1.3242ptEmployed/Other	112 (77.78%)		
Household income			
Low Income (< $25k)	103 (71.53%)		
Middle Income ($25k-$74k)	31 (21.53%)		
High Income (≥$75k)	5 (3.47%)		
Prefer not to answer	5 (3.47%)		
Household size			
1	44 (30%)		
2	57 (39%)		
3	27 (18%)		
4 or more	16 (13%)		
Children in household			
Have at least one child under 18	16 (15%)		
	Baseline	Post Intervention	p-value for change
SNAP participation			
Yes	76 (52.78%)	63 (43.75%)	0.0001
Financial strain (I don't have money to pay my bills)			
Never/rarely	29 (19.74%)	26 (18.06%)	0.0006
Sometimes	64 (44.44%)	46 (31.94%)	
Often/always	51 (35.42%)	68 (47.22%)	
Don't know	0	2 (2.78%)	
General health			
Poor	47 (38.52%)	42 (34.71)	0.0001
Fair/good	69 (56.56%)	69 (57.02%)	
Very good/excellent	5 (4.10%)	8 (6.61%)	
Don't know	1 (0.82%)	2 (1.65%)	
Food security status			
Marginal Food Security	2 (1.39%)	1 (< 1%)	0.91
Low Food Security	25 (17.36)	22 (15.28%)	
Very Low Food 46.8-7.5,-1.3242ptSecurity	117 (81.25%)	121 (84%)	
Nutrition security status in the last 12 months, (I/we) worried			
that the food (I was/we were) able to eat would hurt (my/our)			
health and well-being.			
Never/Rarely	51 (35.42%)	46 (31.94%)	0.82
Sometimes	53 (36.81%)	49 (34.03%)	
Often/Always	34 (23.61%)	41 (28.47%)	
Don't Know	6 (4.17%)	8 (5.56%)	
**Systolic BP (mmHg)**	127.035 (25.36)	130.40 (18.24)	0.27
**Diastolic BP (mmHg)**	78 (14.35)	80.23 (12.13)	0.11
**Dietary intake**			
Mean Change Women in F/V cups	0.22		*P* = 0.002
Mean Change Men in F/V cups	0.25		*P* = .002

**Table 4:** Clinical changes pre and post intervention stratified by rural and urban location and SNAP participation.

Clinical Value Change	Mean and 95% CI Pre-Post MTM		Mean and 95% Pre-Post Grocery Rx	
	Rural (*N* = 16)	Urban (*N* = 17)	Rural (*N* = 64)	Urban (*N* = 47)
Systolic blood pressure (mmHg)	−1.26 (-1.85,.51)	−8.25 (-11.2, -.69)^*^	−4.17 ('-7.45,−2.41)^*^	−7.06 (-.9.3,−5.14)^*^
Diastolic blood pressure (mmHg)	-.40 (-1.41,.59)	−11.97 (-17.11,−8.18)^*^	−3.52 (-8.8,−2.43)^*^	−4.31 (-9.68,−3.49)^*^
	Mean and 95% CI Pre-Post MTM		Mean and 95% Pre-Post Grocery Rx	
	Rural SNAP (*N* = 12)	Urban SNAP (*N* = 11)	Rural SNAP (*N* = 33)	Urban SNAP (*N* = 20)
Systolic blood pressure (mmHg)	.52 (-2.93, 1.05)	−12.26 (-19.33, 4.80)	−4.21 (-7.41,−2.61)^*^	−3.81 (-12.38, 5.41)
Diastolic blood pressure (mmHg)	-.51 (-2.95, 1.89)	−11.12 (-19.25, 3.46)	−3.50 (-8.8,−2.4)^*^	−1.99 (-2.44, 4.26)

all models adjusted for race, age, sex, hypertensive medication use, household income, food assistance use, and baseline measure.

all models adjusted for race, age, sex, hypertensive medication use, household income, and baseline measure of systolic or diastolic mmHg.

^*^p < .05.

Table 4. indicates that within the MTM group among rural participants there was no significant change in blood pressure. However, within the MTM group among urban participants, there was a significant decrease in systolic blood pressure (-8.25mmHg; 95% CI−11.2, -.69) and a decrease in diastolic blood pressure (-1.97mmHg; 95% CI−2.11, -.18). In addition, across both rural and urban areas in the Grocery Rx program, there was a significant decrease in both systolic and diastolic blood pressure. Results further examined the effect of SNAP participation in addition to receiving the FIM programs. In general, there was no significant additional effect. However, among rural residents in the Grocery Rx program that also received SNAP (*N* = 33) relative to those not receiving SNAP (*N* = 31) there was a significant decrease in systolic blood pressure−4.21mmHg (95% CI−7.41,−2.61) and diastolic blood pressure−3.50mmHg (95%CI−8.8,−2.4).

### Cost analyses results

The intervention specific cost differences were primarily driven by the cost of food and postage. Participants in the MTM arm of the study received bi-weekly deliveries at a cost of $181 per month inclusive of shipping costs. In the Grocery Rx (urban) arm, participants received a card directly from Soda Health which was allocated $100 each month of the study. The Grocery Rx (rural) arm utilized physical vouchers, also in the amount of $100, which were mailed to participants once per month, incurring additional postage costs and additional staff time to prepare the packages. The Grocery Rx (online) arm utilized online shopping with account credits for $100 each month; in addition to the amount available to purchase groceries, the Instacart arm was provided an additional $10 to cover service fees. While MTM participants received the full value of the meals provided, grocery Rx participants utilized, on average, 84.1% of the funds allocated. Cost calculations include the full allocation provided to participants to avoid understating the potential costs of implementation.

As may be expected, costs of the 3 grocery Rx interventions are similar at less than $400 per participant. There were small differences attributed to the additional funds to cover fees from Instacart (online arm), the additional shipping costs associated with sending vouchers for Food City (rural arm), and the lack of shipping costs covered by Soda Health (urban arm) to send shopping cards to participants. For all the grocery Rx programs, more than 75% of the total costs were available to participants to spend on food, a percentage that would grow as the intervention was extended. The MTM arm of the intervention cost more than the grocery Rx arms at just over $600 for the 3-month period for each participant. The difference between home delivery of 20 frozen meals per month and access to $100 in groceries makes direct comparisons difficult. However, the value of the food and delivery ($543 for 3 months) accounted for 90% of the total costs of that arm of the study.

### Semi-structured qualitative interview results

A total of 30 participants were contacted about participating in interviews, and 20 interviews were completed (67% response rate). The participant sample sizes from each intervention group included: six MTM, six grocery Rx (rural), and eight grocery Rx (urban). Four main themes were identified from the interview data, including: user satisfaction, user recommendations, communication and nutrition education, and health and behavioral outcomes. The main themes were consistent across participants from each of the three groups (MTM, grocery Rx rural, and grocery Rx urban), so the results are presented collectively with relevant divergence among groups noted in Table 5. Participants reported being motivated to participate in the program for the potential health benefits, due to being food insecure/needing food assistance, and because the program was easy to participate in. Table 5. contains detailed descriptions of each theme and representative quotes.

**Table 5:** Participant semi-structured program feedback interview themes.

Theme	Detailed description	Representative quote
**Participant satisfaction**	Participants found the program to be helpful, convenient, easy to use, and educational, and the food provided to be enjoyable. The MTM participants also discussed the convenience of having healthy, heat-and-eat meals delivered.	“*It's healthy food. It's it saves time where you don't have to go out to the grocery store. And it just really helps out, especially if you know if you get up, you got an appointment, you could just put your food in the microwave and eat it and then go.”–MTM Participant*
**Participant recommendations**	The main recommendations participants provided were related to expanding program elements such as having the program last longer than 3 months or increasing benefit amounts. When encouraged to provide detailed feedback, the recommendations were focused on the logistics of the food prescriptions including increasing clarity of the foods allowed to be purchased, sharing recipes that included only covered food, increasing the variety of covered items and meals to choose from, better ways to track the card/voucher balance (for Grocery Rx users), and better scheduling of delivery services to preserve food quality and safety.	“*Well, it would have helped if it had been a little more. But I really sound like I'm selfish when I say that, and I really don't want to say it that way.” – Grocery Rx rural participant*
**Communication and nutrition education**	Participants reported receiving various communications including reminders to use food benefits, health tips, and user/customer support. Participants across FIM groups reported positive perceptions of the communications received during the program, particularly the information on how to use their benefits and how to eat healthier.	“*It just kept me aware of what I had and, you know, what I could get with it, and how I needed to eat, and it really kind of motivated me to eat a little healthier and stuff.” – Grocery Rx urban participant*
**Health and behavioral outcomes**	Participants in all groups reported health outcomes in relation to participation. This included perceived improvements in blood pressure management and other pre-existing health conditions, and changes in food-related behavior, including food choices, grocery shopping, portion control, and cooking methods.	“*I really like the low sodium content [of the food]…So, I was able to actually alter my diet from my usual diet and I felt a lot better just physically.” -MTM participant*

## Discussion

According to process measures and implementation outcomes, our study is impactful in two meaningful ways. Our primary objective was to determine if a screening decision tool allocated individuals into an appropriate FIM program based on needs and preference. Our match rate of over 90% for grocery Rx, among both rural and urban settings, indicates the development, refinement, and use of the screening decision tool was effective. However, the match rate of around 75% for MTM indicates further refinement is still warranted. It is important to note that these findings provide key information about feasibility and fit of our approach, but do not provide clinical evidence of effectiveness of the screening tool relative to other types of allocation methods. There is a large amount of evidence regarding patient decision aids in helping individuals make informed healthcare choices (36). There are also emerging screening tools to identify food (28) and nutrition security status (37). However, to our knowledge there are no evidence-based tools to help patients make informed choices about which food is medicine program to utilize after they have screened positive for food and nutrition insecurity.

In addition, further evidence of the success of our tool is indicated in utilization of the FIM programs. First, our participants redeemed 84% of their grocery benefits and 99.5% of intended meals were delivered to participants in the MTM. Relative to other studies, this is a high redemption rate and completion rate across our lower income rural and urban adults. Other studies have reported a redemption rate ranging from below 20% to 80% or greater, (38) with one study indicating an overall voucher redemption rate of 52% (25). However, our results are similar to other grocery Rx programs, such as the Fresh Rx produce grocery program, which had a rate of 79% (26). The average adult participant spent 73.1% of their produce prescription dollars during program enrollment (the remainder was unspent) (39). A similar FIM program in rural and urban areas of Georgia had a 59% retention rate at 6 months, (40) compared to our engagement and retention rates of 95% and 91%, respectively. One key reason for the high retention and engagement rate is the shorter length of our program being 3-months. However, in general across most states using a FIM program the length is typically 3-months (41, 42). We attribute some of our success with retention and engagement rate to utilizing a screening decision tool to tailor the intervention to participants rather than conducting a conventional randomized controlled trial. In addition, we had dedicated staff to provide high touch patient care and a text help line.

Over 12 weeks in both MTM and grocery Rx programs, there were significant changes in blood pressure among rural and urban adults. While these results are promising, this was not a randomized clinical trial powered to detect change in blood pressure, thus interpretation needs to be cautious. Results are similar to other food is medicine programs which indicate reduction in blood pressure (43). However, our study did not have a comparison group and thus we cannot make any comparisons between our study relative to standard care. Moreover, our study was not powered to detect changes in blood pressure but was designed to seek information related to tailoring a FIM program for user needs, preferences, and geographic location to retain engagement. In our qualitative interviews, participants reported improved symptoms related to cardiovascular disease including reduced swelling, feeling better overall, and being able to manage their blood pressure better. Previous studies have found mixed results of effects of FIM on blood pressure, with some studies finding a reduction in systolic and diastolic blood pressure (39) among adults with HTN utilizing a produce prescription program or a grocery voucher program, (17) while another yielded no change in blood pressure between treatment and control over a 6-month program (41). Our results, coupled with these previous findings, point to the need for larger scale studies utilizing a user-centered approach to allocate individuals based on their level of need, stage of disease, and preferences in order to understand for whom, how long, and in what manner a FIM program can improve clinical outcomes.

Our self-report outcomes indicated strong improvements in self-reported dietary intake, general health, and financial strain. While this is a small change, it is also clinically meaningful (44, 45) and points to how a tailored dietary program can “move the needle” on key health outcomes. Other FIM programs have reported similar findings with general health and dietary intake (39). Our study further expands this research to suggest that a short-term intensive FIM program designed for the user can improve diet and feelings of improved health in the short term. Participants self-reported improved dietary habits such as eating more fruits and vegetables, eating foods that are beneficial for managing chronic conditions like diabetes, and being able to purchase healthful foods or meals they would not be able to purchase without the benefits they were provided.

Our study also explored how SNAP participation, in addition to receiving a tailored FIM program, influenced blood pressure change. Our results point to the effects that dual participation in SNAP and a grocery Rx program can have on reducing blood pressure. While this result was not powered, the study shines a light on the key aspect of dose of a food is medicine program, which can play a key role in improving health outcomes. Given that the overall sample didn't improve clinical outcomes, but those with the additional benefit did improve clinical outcome, it suggests that the food supports from this program in addition to the SNAP benefit might be the ideal amount needed for food insecure individuals to maintain a healthy diet. Studies to date have indicated that SNAP has been associated with unhealthy purchases, (46, 47) while others have indicated that participation reduces food insecurity and lowers annual health care expenditures (48). Our preliminary findings corroborate previous findings suggesting that, in the short-term, SNAP + FIM can ease financial strain, reduce stress, and free up cognitive bandwidth, which all in turn allows individuals to make healthier choices (48). A recent scoping review indicated that nutrition education + monetary incentives among SNAP participants resulted in the greatest improvements for dietary intake (49). Our results further support the notion of offering SNAP participants an additional supplemental program for purchasing healthy food that meets their individual needs. This can allow participants an opportunity to free up financial resources while also engaging in a program that allows for learning how best to utilize their SNAP benefit in a healthful manner (50).

Our study had several limitations worth noting. Participation was only for 3 months, limiting the ability to assess long-term effects. In addition, there was no tailoring based on the level of food security and disease management. This is a critical piece in helping to inform who and at what level these clinical-community linked resources are most appropriate. Participants most likely reported improved measures on self-report outcomes due to social desirability and may not reflect actual changes in dietary intake. For some participants, the health metrics provided by clinics did not coincide with the end of the program (3 months post-baseline). As a result, health measures for post-intervention were self-reported by 17 participants. There was a significant lag-time between completing the intervention and receiving post program blood pressure measurement. This most likely resulted in blood pressure readings regressing to the mean. Moreover, qualitative interviews were only conducted with program completers who volunteered to do an extra interview and thus may have had a more favorable perception of the program than others who did not complete the program or did not want to participate in additional data collection. However, nearly all participants completed the entire study, and the qualitative sample had a high participation rate (67% of those invited to do an interview participated), lessening these concerns about qualitative sampling.

## Conclusion

Our feasibility study utilizing a screening decision tool to allocate individuals to a tailored food is medicine program resulted in high patient engagement, improved dietary intake, and, for certain populations, reductions in blood pressure. Future studies need to incorporate a user-centered framework within the development and testing of food is medicine interventions to further understand for whom and in what manner these types of programs are effective at improving clinical outcomes.

## Data Availability

The datasets presented in this study can be found in online repositories. The names of the repository/repositories and accession number(s) can be found below: http://datadryad.org/share/LINK_NOT_FOR_PUBLICATION/94drrgBffY8BL8tjaGkyzUxrIMvBiat7afW-ThN_GTc
